# Preparing hospitals and health organizations for AI: practical guidelines for the required infrastructure

**DOI:** 10.3389/fdgth.2025.1605006

**Published:** 2025-08-18

**Authors:** Emil Byberg, Marco Crimi

**Affiliations:** ^1^ R&D Office, Net-Medicare SRL, Bergamo, Italy; ^2^ Biomed Research Office, Kaleidos SCS, Bergamo, Italy

**Keywords:** artificial intelligence, CDSS, digital transformation, enterprise architecture, IT governance, knowledge management, CFIR

## 1 Introduction

AI is rapidly transforming the medical field: From predictive algorithms to enhanced robotic surgeries, there is relatively wide potential for AI to improve clinical outcomes (1–3). Yet, evidence suggests that AI's capabilities cannot be fully realized without first ensuring a solid foundation, be it technological, educational, clinical, or ethical. Previous studies have reported that AI implementation without adequate infrastructure leads to inefficiencies and gaps in its development, execution, and monitoring (4, 5), and according to the literature, hospitals tend not to possess the mentioned infrastructure (6, 7).

Healthcare organizations tend to rush to adopt AI technologies (8, 9), and this pattern mirrors historical health IT adoption challenges where technology outpaced organizational readiness. This can lead to challenges like poor AI performance, data silos, regulatory compliance issues, and privacy risks (10), as well as compromising patient safety, eroding trust in AI systems, delaying the r AI's benefits, and resulting in wasted resources (11–13).

Hence, healthcare organizations are recommended to prioritize the development of a robust infrastructure before integrating AI. With a list of requirements and how to achieve them, healthcare organizations can create an environment where AI reaches its potential.

This Opinion paper answers the following research question: According to the latest findings, what guidelines should healthcare organizations follow to increase their chance of optimal AI deployment? The paper aims to (1) discover cross-departmental foundational requirements that influence AI adoption and (2) elaborate strategies for achieving such requirements.

## 2 Methodology

To meet its goals, this paper has a two-step methodology to first define the requirements and secondly to discuss the strategies to achieve them:

1. Being a group synergetic, cross-departmental frameworks more effective than single generic one (14, 15), this paper uses the Design Science Research (DSR) practical approach to problem solving (16, 17) to find and choose said frameworks with a focus on highlighted digital transformation themes (18–20):
  ○ Organizational alignment and integration.
  ○ Accountability and decision-making.
  ○ Accessibility and usability of data.
  ○ Collaborative learning and knowledge transfer
  ○ Improved clinical practice
2. To discuss what strategies achieve these goals, this paper employs the Consolidated Framework for Implementation Research (CFIR), a proven methodology to evaluate the various factors that influence the implementation of health interventions (21, 22). Its five domains are:
  ○ Intervention Characteristics.
  ○ Outer Setting.
  ○ Inner Setting.
  ○ Characteristics of Individuals.
  ○ Implementation Process.

## 3 Key infrastructure frameworks for healthcare organizations

To analyze the five digital transformation themes mentioned above, the respective chosen frameworks according to DSR are:

• Enterprise Architecture (EA)
• IT Governance
• FAIR Principles and Standardization
• Knowledge Management & Knowledge Sharing (KM-KS)
• Clinical Decision Support System (CDSS)

### 3.1 Enterprise architecture (EA)

EA aligns an organization's processes, information systems, and infrastructure with its business goals. In healthcare, EA ensures interoperability, data standardization, and seamless integration of emerging technologies. Enterprise Transformation Projects (ETPs) are large-scale initiatives that leverage EA to modernize healthcare systems, optimizing workflows, enhancing data governance, and fostering innovation (23). Successful implementations often use phased approaches to minimize disruption to clinical operations. By implementing robust EA through strategic ETPs, healthcare organizations can systematically transition toward AI-ready infrastructures, improving efficiency and patient outcomes as well as understanding the impact of their digital interventions, as demonstrated by real-world applications (24).

Enhancements in data governance frameworks within EA can bolster the credibility of AI-driven decision-making processes in healthcare, ensuring that AI systems are transparent and accountable (25). A robust EA also has a well-defined set of strategic requirements that describe data management and AI integration in process terminology, allowing both high-level management and operational staff to better understand and appreciate the technological implications of AI, especially when paired with internal educational initiatives (26).

### 3.2 IT governance (ITG)

ITG ensures ethical practices, regulatory compliance, and alignment with clinical objectives when integrating new technologies. ITG emphasizes patient privacy and data security throughout technological implementations, especially in new patient-data management settings like clinical AI (27). Effective ITG establishes clear accountability for AI system performance across the entire lifecycle, from development to execution. With increased emphasis on transparency and data management, ITG not only fosters innovation but also mitigates risks associated with AI deployment.

Modern ITG recommended practices highlight AI pre-deployment activities and checkpoints as crucial to successful innovation, with data governance compliance, algorithmic validation, post-deployment monitoring, and stakeholder engagement as beneficial for successful, transparent, accountable, and continuous surveillance of AI (28). These practices help identify potential biases, ensure model generalizability across patient populations, and maintain audit trails for regulatory compliance. Governance frameworks are also beneficial in detecting safety alerts that might arise post-deployment (29).

### 3.3 FAIR principles and standardization

Established data standards are crucial for ensuring that AI training and execution in operational healthcare contexts are accurate, secure, and ethically compliant, and the FAIR Principles (Findable, Accessible, Interoperable, Reusable) describe how data should be structured to enable such features (30, 31). With a focus on standardization, multiple frameworks embody these principles to provide practical guidance towards greater FAIRness of datasets, such as FHIR, the FAIRification Framework, the MDA Framework, and the FAIR Hourglass, among others (32–35).

FHIR (Fast Healthcare Interoperability Resources) is a proven framework widely used in health IT that builds on FAIR by providing a standardized, web-based framework for data exchange (36). FHIR's modular resources enhance interoperability, enabling seamless integration across systems while maintaining FAIR compliance. By leveraging RESTful APIs (Application Programming Interfaces) and structured data formats, FHIR facilitates real-time access and machine-readable health records, supporting AI-driven analytics and precision medicine (37).

### 3.4 Knowledge management and knowledge sharing (KM-KS)

KM-KS frameworks focus on the efficient capture, organization, and dissemination of knowledge within an organization (38). Real-world evidence suggests that effective KM-KS leads to enhanced decision-making, better patient care outcomes, and effective healthcare operations (39, 40). KM-KS frameworks have a close relationship with organizational culture, with interpersonal trust in sharing and receiving knowledge among healthcare staff members having a pivotal role. Hence, effective KM-KS requires not only workflows that facilitate knowledge exchange but also initiatives directed towards team building and training (41, 42).

Advancements in AI need robust knowledge sharing within the workplace, as higher knowledge means greater skills in handling AI, hence improving productivity in the context of AI technologies (43). Knowledge infrastructure encompasses the organizational culture, structure, and technological resources, supporting the idea that integrating KM-KS is an interprofessional collaborative learning process (44).

### 3.5 Clinical decision support systems (CDSS)

CDSS can be described as frameworks that define how to effectively design operational systems that support healthcare providers to make more informed clinical decisions by leveraging patient data and clinical guidelines (45, 46). Providers can benefit from AI to enhance their functionalities withthe analysis of vast datasets to provide tailored and case-specific recommendations, significantly improving clinical efficiency and patient outcomes (47, 48).

However, evidence underlines the necessity of understanding when and where AI-CDSS systems should be deployed to maximize their impact based on pre-existing well-designed CDSS (49). Effective CDSS tailored to clinical scenarios should be developed in collaboration with domain experts before the integration of AI (50). Adapting AI-CDSS to fit the daily clinical tasks of healthcare workers is relatively complicated, showing that a lack of proper preparatory alignment can adversely affect user experience and system efficacy (51).

## 4 Discussion on achieving an AI-ready infrastructure

This section of the paper discusses the strategies that emerge from reinterpreting the scientific literature presented in the previous section with the five CFIR domains. Each of the following sub-sections discusses the theme dealt with, its context, and three strategies towards it.

Table 1 extends this discussion and presents more in-depth actionable items for each strategy.

**Table 1:** Themes, strategies, and actionable items towards successful AI implementation.

Themes	Strategies	Actionable items
Digital infrastructure	Evaluate the current digital infrastructure	Assess existing hardware, software, and connectivity
		Engage stakeholders across departments to gather insights on needs
	Identify gaps and areas for improvement	Analyze assessment results to find deficiencies
		Create a prioritized list of infrastructure upgrades
	Implement infrastructure upgrades in phases	Develop a phased implementation plan that minimizes disruption to operations
		Ensure new technologies possess features enabling seamless integration with existing workflows
Data governance frameworks	Develop robust data governance policies	Draft comprehensive policies outlining data usage, privacy, and compliance
		Involve multidisciplinary teams in policy creation for inclusivity.
	Educate staff on data governance principles	Facilitate training sessions for personnel on data governance protocols
	Regularly review and update governance policies	Schedule routine evaluations of data governance policies to ensure alignment with evolving technologies and regulations
Skilled workforce development	Establish ongoing training programs for AI technologies	Develop and implement training sessions focused on role-specific applications of AI
		Utilize online platforms for accessible training resources
	Foster interdisciplinary learning and collaboration	Create opportunities for healthcare professionals to engage in knowledge-sharing initiatives
		Set up communities of practice to promote collaborative learning
	Encourage participation from all staff levels	Design programs that are inclusive and cater to various professional roles
		Highlight the benefits of AI technologies for different departments in communications
Collaborative partnerships	Create collaborative partnerships with stakeholders	Identify and engage with external partners, including technology firms and academic institutions
		Leverage these collaborations to share resources and expertise
	Form multidisciplinary teams for effective implementation	Assemble teams consisting of clinicians, data scientists, and external advisors to guide AI integration
		Promote teamwork through clear communication and shared goals
	Engage in stakeholder forums for knowledge exchange	Participate in collaborative networks and forums to gather insights and address common challenges
		Encourage the sharing of best practices among healthcare organizations
Continuous evaluation frameworks	Implement structured feedback mechanisms	Set up systems that enable healthcare providers to report their experiences with AI technologies
		Facilitate both qualitative and quantitative feedback collection
	Regularly analyze performance metrics of AI systems	Utilize performance data to gauge the effectiveness of AI tools in real-world applications
		Compare metrics against established benchmarks to identify improvement areas
	Adapt AI technologies based on user input	Create processes for iterating and refining AI solutions based on feedback
		Ensure that AI systems remain responsive to the needs of healthcare providers and patients

### 4.1 Digital infrastructure

A solid digital infrastructure facilitates interoperability, data standardization, and seamless integration of emerging technologies. The successful deployment of AI technologies often hinges on a thorough understanding of existing infrastructure gaps. This aligns with the “Inner Setting” and “Intervention Characteristics” domains, emphasizing the importance of organizational readiness for new interventions.

Recommended strategies:

• Assess the current digital infrastructure, identifying gaps in hardware, software, and connectivity that may impede AI integration
• Engage stakeholders across departments to gather insights on technological needs and preferences.
• Implement phased upgrades to minimize disruptions during the transition. Ensure that new technologies can seamlessly integrate with existing workflows, with the organizational goals in mind.

### 4.2 Data governance frameworks

Establishing robust data governance frameworks is essential for managing data privacy, security, and ethical use. These frameworks ensure that the data used for AI systems aligns with regulatory requirements and organizational values. This aligns with the “Outer Setting” and “Inner Setting” domains, emphasizing the role of external regulatory pressures alongside internal organizational policy and culture.

Recommended strategies:

• Develop robust data governance policies that clearly outline principles of ethical data use, compliance with regulations, and protection of patient privacy and security.
• Facilitate training sessions for staff on these policies in the context of AI integration, enhancing organizational understanding of data governance implications.
• Regularly review and update these policies to adapt to evolving AI technologies and regulatory standards, ensuring transparency and accountability in data management.

### 4.3 Skilled workforce development

A skilled workforce is vital for the successful utilization of AI technologies in clinical settings. Continuous development and training address the needs of healthcare professionals and facilitate the adoption of new technologies. This aligns with the “Characteristics of Individuals” and “Inner Setting” domains, focusing on staff competencies and their perceptions of AI technologies.

Recommended strategies:

• Establish ongoing training programs on both technical skills and practical AI applications tailored to specific roles.
• Promote interdisciplinary collaboration through knowledge-sharing initiatives and communities of practice, enhancing team skills and a culture of continuous learning.
• Encourage participation from all levels of staff in training programs to help them recognize the value of AI and its potential to improve clinical practices in their everyday roles.

### 4.4 Collaborative partnerships

Forming collaborative partnerships enhances the support system surrounding AI implementation in healthcare. These partnerships allow organizations to leverage external expertise, share resources, and establish best practices. This aligns with the “Outer Setting” and “Implementation Process” domains, which consider how external factors impact organizational changes.

Recommended strategies:

• Create partnerships with external stakeholders, including technology firms, academic institutions, and regulatory bodies, to facilitate the sharing of resources and best practices.
• Form multidisciplinary teams that include clinicians, data scientists, and external partners to enhance the development and implementation of AI systems tailored to specific healthcare needs.
• Engage in stakeholder forums and collaborative networks to pool knowledge, resources, and strategies that address challenges associated with AI implementation.

### 4.5 Continuous evaluation frameworks

Continuous monitoring and evaluation are crucial for identifying areas of improvement and ensuring that AI systems remain effective and relevant to clinical needs. This aligns with the “Implementation Process” domain, which emphasizes the systematic evaluation of interventions to foster successful integration and responsiveness to user feedback.

Recommended strategies:

• Implement structured feedback mechanisms that facilitate monitoring of AI systems' performance and user experiences.
• Use qualitative and quantitative metrics to gauge AI effectiveness and identify areas for improvement based on real-world applications.
• Adapt the AI technologies based on feedback and evaluation results, ensuring responsiveness to the needs of healthcare providers and maximizing the systems' efficacy and safety.

## 5 Conclusion

Due to industry trends, healthcare organizations are at risk of suboptimally implementing AI. Evidence suggests a knowledge gap in high management and the need for practical guidelines for digital transformation stakeholders. The authors' reinterpretation of recent literature suggests the presence of five actionable areas, each with strategies that can guide stakeholders in moving towards scientifically supported AI implementation. If acted upon, these strategies provide a foundation for optimal AI deployment.

This paper demonstrates that recent scientific literature holds valuable knowledge for healthcare management and that more in-depth studies should be performed to promote infrastructural prioritization over AI. Being this an Opinion type of paper with a general focus, its main limitation is its lack of depth in the quality and quantity of strategies elaborated, pointing towards focused investigations on the implications of its outcomes, as well as a study on why the suboptimal behaviour exists despite the scientific evidence, as potential future research.

For patients to access the health benefits AI can provide, infrastructures are essential. Through practical guidelines, research can support preparing hospitals and health organizations for AI.
